# A Bourdieusian approach to class‐related inequalities: the role of capitals and capital structure in the utilisation of healthcare services in later life

**DOI:** 10.1111/1467-9566.13028

**Published:** 2019-11-25

**Authors:** Ivana Paccoud, James Nazroo, Anja Leist

**Affiliations:** ^1^ Institute for Research on Socio‐Economic inequalities (IRSEI) University of Luxembourg Luxembourg City Luxembourg; ^2^ Cathie Marsh Institute for Social Research University of Manchester Manchester UK

**Keywords:** inequalities, class, Bourdieu, capitals, healthcare utilisation

## Abstract

This paper draws on Bourdieu's theory of economic, social and cultural capital to understand the relative effect of the volume and the composition of these capitals on healthcare service use in later life. Based on data from the fifth wave of the Survey of Health, Aging, and Retirement in Europe (n = 64,840), we first look at the contribution of each capital in the use of three healthcare services (general practitioner, dentist and hospital). Using cluster analysis, we then mobilise Bourdieu's concept of habitus to explain how the unequal distribution of material and non‐material capitals acquired in childhood lead to different levels of health and hospital care utilisation in later life. After controlling for demographic and health insurance variables, our results show that economic capital has the strongest individual association among the three capitals. However, the results of a cluster analysis used to distinguish between capital structures show that those with high non‐material capital and low material capital have higher levels of primary healthcare utilisation, and in turn lower levels of hospital use. Bourdieu's approach sheds light on the importance of capitals in all forms and structures to understand the class‐related mechanisms that contribute to different levels of healthcare use.

## Introduction

Persistent socioeconomic inequalities in health that continue into later life alongside the ageing of the population have important implications for population health in contemporary Europe, as elsewhere. This is especially so in the light of consequently rising demands for health services and the possibility of existing inequalities in the ability to access appropriate healthcare. Seeing a doctor or a health professional on a regular basis is considered to be a vital investment in health. Indeed, appropriate use of healthcare can facilitate the early diagnosis of health problems, making treatment easier and less costly (World Health Organisation 2019). Appropriate healthcare becomes especially important later in life, when there may be increasingly complex health needs.

Much of the literature on socioeconomic inequalities in the use of healthcare services situates income as a main predictor of healthcare utilisation, and distinguishes between pro‐poor and pro‐rich healthcare services (Allin 2008, Morris *et al*. 2005). However, measures of socioeconomic status provide descriptive classifications of socioeconomic inequalities but offer limited insights into how and why such inequalities exist (Connell and Connell 1977). Moreover this may particularly be the case for older people, as traditional ways of measuring socioeconomic status via income, education and occupation are even more problematic once people retire from paid employment (Allin 2008, Nazroo 2017).

Previous research on healthcare access draws on the dominant behavioural model of health service use (Andersen 1995). This model points to the importance of three factors: those that are predisposing (individual characteristics such as age, gender, education and personal beliefs), enabling (structural and system‐level characteristics such as income and healthcare insurance) and needs related such as self‐perceived health or the degree of illness (Bremer *et al*. 2018, Dey and Jorm 2017, Lo *et al*. 2016). Numerous studies have also looked at the access dimension of the use of healthcare services through the notions of availability, accessibility and affordability of healthcare services (Ensor 2004, Harris *et al*. 2011, Kyriopoulos *et al*. 2014, Yamada *et al*. 2015). Others claim that only health status predicts the use of healthcare services, and that this should be the most important factor in healthcare use (Goodwin and Andersen 2002, Parslow *et al*. 2004). However, these approaches neglect accumulated class‐related individual resources that translate into health literacy and health prevention practices, which help individuals to optimise their use of healthcare services.

In this article, we argue that Bourdieu's capital‐based approach can serve as an important complement to the current understanding of the determinants underlying health inequalities in the use of healthcare services. What is interesting about his approach is that it is broad enough to consider not only the monetary dimension of capital but also other forms of capital (social and cultural), represented in lifestyle indicators, educational prestige and the symbolic dimensions of class relations, such as social relationships. This approach enables us to derive specific predictions based on the possession of different forms of capital. By looking into the relative contribution of each of the capitals and the effects of different compositions of capitals, we aim to contribute to a broader understanding of the class‐related mechanisms of healthcare access in later life. This is especially important as studies have shown that ageing populations have been rather neglected in research on class and health (Nazroo 2017). We begin by providing a more detailed summary of Bourdieusian approaches to understanding capital.

### Bourdieu's forms of capital

According to Bourdieu, social class is a social group defined relationally in social space by its possession and utilisation of various capitals such as economic, cultural and social capital (Bourdieu 1986):

☐ *Economic capital* accounts for money, property and other financial assets such as bonds, shares and stocks. In the health domain, economic capital refers to the material resources required to reach, pay in advance for standard health services, and purchase better services (complementary health insurance, treatments abroad, etc.).
☐ *Cultural capital* exists in three forms: in the embodied state (through behaviours and dispositions learned over the life course), in the objectified state (through cultural goods the individual possesses such as books or musical instruments) and in the institutionalized state (such as an educational qualification). Bringing this into the health field, the concept of cultural health capital reflects individual skills, verbal and non‐verbal competences, interaction styles, and attitudes and behaviours, that relate to health practices, including the use of health services (Shim 2010). It also plays a crucial role in the unequal production and distribution of health (Abel 2007).
☐ *Social capital* is defined by Bourdieu as the “aggregate of the actual or potential resources which are linked to the possession of a durable network of more or less institutionalized relationships of mutual acquaintance and recognition” (Bourdieu 1986: 248). In relation to the use of healthcare services, an individual's social network can be drawn on to access health information and support, speed up access to health care, or get recommendations on good quality healthcare providers. Song (2011), for example, shows that social capital has direct, indirect and intervening effects on the social reproduction of health, such as through providing a valuable information network, emotional support and encouraging engagement in healthy behaviour, among other things.

Bourdieu's approach is particularly useful to study inequalities as it relies on three essential ideas capturing the mechanisms that lead to the unequal use of healthcare services. Firstly, there is the idea that the volume of capitals possessed is linked to particular dispositions or practices, secondly the fact that the capitals can be transmitted across generations, and thirdly, that it is not only the amount of a particular capital individuals possess that matters but also how this relates to the “structure of their capital, that is, […] the relative weight of the different types of capital in the total volume of their capital” (Bourdieu 1998: 7). We will elaborate on each of these ideas in turn.

For Bourdieu, people are distributed in the social space based on two dimensions: the volume of each capital and the relative amounts of the different types of capital they possess – the structure of their capital. Those with a similar volume of the capitals and a similar capital structure thus occupy a similar social position in the social space: “all agents are located in this space in such a way that the closer they are to one another in those two dimensions the more they have in common; and the more remote they are from one another, the less they have in common” (Bourdieu 1998: 6). Each of these social positions then correspond to a “*habitus,”* a fundamental concept in Bourdieu's theoretical framework. Habitus is an embodiment of the social and material conditions in which people live which generate distinctive tastes, preferences and lifestyles that are then translated into social practices. The notion of habitus is then related to an individual's “*choice of necessity”* in everyday life (Bourdieu 1998). Hence, the practices of individuals with a low volume of capitals are constrained to what is necessary, which leads to adaptation and acceptance of this necessity (Bourdieu 1984). Inspired by this line of reasoning, it has been pointed out that “capitals are resources for engaging in strategic actions that are intended to position their holders advantageously relative to the other members of social systems” (Veenstra and Abel 2019: 1). For instance, those with high levels of all capitals have the freedom of choice that allows them to make more informed, long‐term and strategic decisions about their health, and in turn create distinctive practices that can translate into a health advantage. This is further highlighted in a study by Smith and Dumas (2019) which shows that Bourdieu's concept of “practical sense” helps us understand how individuals internalise priorities at the expenses of others. Furthermore, in their study of the disposition of underprivileged young women towards weight control strategies, Dumas *et al*. (2014) found that despite the participants’ health and weight knowledge, their practices were influenced by their immediate needs and responsibilities. Applying Bourdieu's concept of necessity in our study, it could be assumed that people with lower stocks of the capitals will only use healthcare services out of medical necessity – when ill for example – while those with higher capital levels will utilise them more strategically – as a form of health prevention for example.

Another important element of Bourdieu's framework is the *intergenerational transmission* of capital. While this is widely acknowledged for economic capital (in the form of wealth), distinctive forms of knowledge, skills and abilities are also transmitted from one generation to the next (Bourdieu 1986). It is within the family and school contexts that individuals develop their verbal skills and acquire their first health habits, such as having a healthy diet or visiting the dentist and doctor regularly. For example, Missinne *et al*. (2014), found that those who used prevention services as children had higher cultural capital and a higher probability of using prevention services in later life. At the moment of the appointment with a health professional, cultural capital may further facilitate interactions by providing credibility and helping individuals to comprehend and express complex medical terms and follow instructions on self‐care (Dubbin *et al*. 2013, Shim 2010). Bourdieu's conceptualisation of social capital also has an intergenerational component through the extension of social connections fostered by parents to their children. This is evident in Moore's research highlighting that the higher someone's initial social position, the higher the quality and quantity of resources reached through one's social connections (Moore *et al*. 2014). An increasing number of scholars are adopting Bourdieu's conceptualisation of social capital which emphasises the importance of network‐based resources that a person can draw on and benefit from (Carpiano 2006, Moore *et al*. 2014, Song 2011, Legh‐Jones and Moore 2012, Stephens 2008).

Finally, for Bourdieu, people are distributed in social space on the basis of a similar composition of the capitals they possess, even within the dominant classes (Bourdieu 1986). For example, Bourdieu distinguishes between those with high economic capital but low amounts of the other capitals (the industrial and commercial elite), those with high cultural capital but lower economic capital (professors, public‐sector executives, artists) and those with high economic and non‐economic capital (private sector executives, doctors and lawyers) (Bourdieu 1986). It is self‐evident that economic capital has a direct impact on health through the environment you live in and through consuming and purchasing premium or supplementary health insurance plans. Those with high wealth can secure comfortable housing in an affluent area free of toxins and pollution, with good transport links and easy access to good quality health services. However, this might not be enough to guarantee the regular use of healthcare services. Instead, an important factor for the regular use of health care may be non‐material capitals (cultural and social capital), that can provide individuals with established healthy habits, access to valuable health information, knowledge of the importance of timely and regular health visits, and strong motivations in staying healthy. Indeed, in his book ‘*Distinction’*, Bourdieu states that health‐oriented practices are adopted by culturally rich fractions in the middle and the dominant classes (Bourdieu 1984).

In the study of health inequalities, Bourdieu's capitals have been used to empirically unveil the class‐related mechanisms producing inequality in health outcomes, such as mortality, physical health and mental health (McGovern and Nazroo 2015, Nazroo 2017, Pinxten and Lievens 2014, Veenstra 2007, Veenstra and Patterson 2012). For instance, Bourdieu's lens has proven helpful in explaining the use of mammography screening, where cultural health capital accumulated early in life was an independent predictor of attending a mammography screening in later life, even after controlling for socio‐economic position (Missinne *et al*. 2014). The research presented here advances the use of Bourdieu's capitals in the health domain in two ways. First, it investigates the relative importance of the three types of capitals in the use of healthcare services. Second, it goes one step further in exploring the different capital compositions an individual possesses, and how differences in composition are related to the utilisation of health and hospital services. The idea tested here is that certain combinations of these capitals – called capital structures – are associated with higher healthcare utilisation.

### Research questions

Using Bourdieu's approach to capital, we will examine:

1. The relative importance of each form of capital on the use of different health and hospital services, and;
2. Whether different capital structures at the individual level explain different utilisation patterns of health and hospital services in later life.

In this article, we use the following definition of health services utilisation: “the process of seeking professional health care and submitting oneself to the application of regular health services, with the purpose to prevent or treat health problems” (Scheppers 2006: 326). In addition, in order to understand the links between socioeconomic inequalities and healthcare utilisation rates, a useful concept is horizontal equity in the use of healthcare services. This reflects the idea that persons, given equal medical needs, will use healthcare services equally, irrespective of their socio‐economic status (SES), so the “equal access for equal need” principle. The alternative, vertical equity, which is the extent to which people with greater health needs are better treated has been shown to be difficult to measure (Sutton 2002, Wagstaff and van Doorslaer 2000), so will not be covered here.

## Methods

### Dataset and variables

We used the fifth wave of the Survey of Health, Aging, and Retirement in Europe (SHARE). SHARE is a panel data on health, socioeconomic status and social and family networks. The data resource profile can be found in Börsch‐Supan *et al*. (2013). It has a cross‐national and multidisciplinary approach that conveys a comprehensive picture of individual and societal aging. The survey is based on probability samples with full population coverage, giving a representative sample of 64,840 individuals, aged over 50, and covering 15 European Countries (Austria, Belgium, Czech Republic, Denmark, Estonia, France, Germany, Italy, the Netherlands, Slovenia, Spain, Sweden, Switzerland, Luxembourg, including Israel). The sampled population are individuals born 1962 or earlier, and their partners/spouses regardless of their age. Except for the variables on income, assets and consumption expenditure, the missing data for the other variables are relatively low (less than 5%). However, this varies across countries. Denmark and Sweden have the lowest percentage of missing data (less than 10 percent), and countries such as Spain, Slovenia, Luxembourg and Israel have considerably higher missing values on wealth variables, reaching over 60 percent on some wealth components.

### Measurement of the use of healthcare services and overnight hospital stays

Access to healthcare services included consultations with a health professional, visits to dental services, and overnight stays in hospital. For consultations we used answers to the following questions: ‘In the last 12 months, about how many times in total have you seen or talked to a medical doctor or qualified nurse about your health? Please exclude dentist visits and hospital stays, but include emergency room or outpatient clinic visits. To simplify the interpretation, we created a binary variable indicating if a person had seen a health professional or not in the last 12 months. Dentist visits were measured through the answer to the following question: “During the last 12 months, have you seen a dentist or dental hygienist?” (yes/no). In terms of hospital stays, the exact question is: “During the last 12 months have you been in the hospital overnight? Please consider stays in medical, surgical, psychiatric or in any other specialized wards” (yes/no), and “How many times have you been a patient in a hospital overnight during the last twelve months?” (count variable). We used the binary and the count variable to investigate the likelihood of having had an overnight hospital stay and the number of stays.

### Covariates

As we were interested in testing horizontal equality, we controlled for an individual's need for health services. This was captured by self‐perceived health status, rated on a five‐point scale (excellent, very good, good, fair and poor). We chose this measure as studies have considered it to be in accordance with the health records of health providers, as well as a valid health status indicator for population health monitoring and a reliable predictor for GP and hospital use among older adults (Bremer *et al*. 2018, Miilunpalo *et al*. 1997, Ritter *et al*. 2001, Vaillant and Wolff 2012; Weinberger *et al*. 1986). It also has an advantage in terms of capturing the biological, psychological and social dimensions of health, as opposed to only the medical diagnosis (Miilunpalo *et al*. 1997). Other covariates included: age, gender, marital status and migration status (born in the country of interview/not born in the country of interview). We also controlled for healthcare factors such as having a national or supplementary health insurance (yes/no). To account for differences in healthcare systems between countries, we adjusted for country of residence.

### Economic capital index

In order to operationalise Bourdieu's notion of economic capital, we used total household wealth. It has been shown that in later life, income and occupational status lose their significance, and wealth (i.e., accumulated economic assets) becomes a more important measure of economic capital (Alessie *et al*. 1997, Miilunpalo *et al*. 1997). Household wealth was calculated as a summary of all assets (the value of home and other real estate assets, minus any mortgage, owned share of own business, owned cars and the values of financial assets – bank accounts, government and corporate bonds, stocks, mutual funds, individual retirement accounts and contractual savings for housing and life insurance policies owned by the household) minus liabilities (Allin *et al*. 2009, Maskileyson 2014). Non‐Euro values were converted. Wealth values of the year preceding the interview were adjusted for purchasing power parity in the same year. To eliminate cross‐national differences in levels of wealth, we use the wealth percentile ranks for each country. This transformation ranks individuals according to their wealth at the national level. Given that for Bourdieu cultural, economic and social capital translate into one's social status, this indicator treats economic capital as a positional good. As 46.6% of the values on the wealth variable were missing, the missing‐at‐random assumption was likely violated. Therefore, we used the study derived imputed wealth values as the most appropriate method to adjust for selective non‐reporting. The imputation model in SHARE is based on the Fully Conditional Specification method “which uses a multivariate sequential approach in an attempt to preserve the correlation structure of the imputed values” (Van Buuren *et al*. 2006). More details on this imputation technique can be found in the SHARE release guides (SHARE Release guide 6.1.1).

### Cultural capital index

Bourdieu's notion of cultural capital brings together three aspects: the institutionalized state, the objectified state and the embodied state. These three dimensions of cultural capital were operationalised in the following way:

☐ To measure institutionalised cultural capital, we utilised respondents’ highest level of education, in line with previous studies (Pinxten and Lievens 2014, Veenstra 2007). We used the 1997 International Standard Classification of Education (ISCED‐97) provided by SHARE. The respondents were asked: “what is your highest educational degree obtained?” and “which degree of higher education do you have?” To ensure cross‐national comparison, each country leader asked a local expert to map the variable into the ISCED‐97. As with the economic capital, we used a relative measure of education that represents it as a positional good. The term “positional good” treats the value of educational qualifications as related to their relative scarcity in the population (Shavit and Park 2016). This was operationalised by subtracting the country‐level average educational qualification from the respondent's educational qualification score.
☐ To measure the embodied state of the cultural capital we composed a parent education variable. First, the variable measuring “mother's education” and “father's education” were standardised with the z‐score and then averaged to create a variable “Parent education.” Parental education was not made relative to the country as we used it as a proxy for the social environment in which the respondent was raised.
☐ Finally, the objectified state of cultural capital was measured through the number of books in childhood which was assessed with the question, “Approximately how many books were there in the place you lived in when you were 10? Do not count magazines, newspapers, or your school books”. The number of books in childhood, has been shown to capture the objectivised cultural health capital well (Missinne *et al*. 2014). At the end, standardised z‐scores were derived for each variable (country specific respondent's education, parent's highest education and number of books in the childhood) and averaged to provide a cultural capital index.

### Social capital index

SHARE participants were asked whether they participated in the following social activities in the last 12 months: (i) participation in voluntary or charitable programmes, (ii) participation in political or community related organisations, (iii) going to a sport, social or other kind of groups and (iv) no participation in any activities. The final score thus ranged from 0 (no participation in any activity) to 3 (participation in all three types of activities). As stated earlier, and as McGovern and Nazroo (2015) note, networking activities are used widely in the health research literature as a proxy measure of peoples’ social connectedness as it has been found to be linked to individual health (Carpiano 2006, Giordano and Lindstrom 2010, McGovern and Nazroo 2015, Song 2011).

### Capital structure

In order to assess capital structures, we used a cluster analysis to classify individuals into groups according to both the type of capital they possessed and the level of capital they possess. To do this, we ran a K‐means cluster analysis with squared Euclidean distance and Ward's algorithm to group people into individual clusters based on different volumes of capitals and their overall composition at the individual level. Again, to ensure comparability between scales, the variables were z‐standardised. Drawing on Bourdieu's idea that people's practices are not solely determined by material factors, we decided to compare non‐material (combined social and cultural, what we call sociocultural) capital against material (economic) capital and averaged the z‐standardised social and cultural capitals.

### Statistical methods

Depending on the outcome, we used generalised linear models with two different regression analyses to examine the association between our independent variables and health outcomes. In the analysis that investigated the association between different types of capitals and capital structure on seeing or talking to a health professional, dentist visits and overnight hospital stays, we used a binary logistic regression. For the number of nights spent in a hospital, we used binominal negative regression as it is used to model over‐dispersed discrete count variables. Odds ratios (OR) with 95% confidence intervals (CI) were calculated for all respondents.

In order to assess the relative contribution of each of the capitals on the outcome variables we added the three capitals simultaneously into the regression models. This strategy was preferred as it captures the way in which capitals build on one another (Pinxten and Lievens 2014, Veenstra 2007). We then ran identical models using our measure of capital structures. Both sets of models controlled for the demographic factors gender, age, marital status, migration status, country of residence, as well as self‐perceived health, additionally adjusting for owning basic health or supplementary insurance coverage. Results stratified by gender were practically similar so we report the pooled analyses.

## Results

### Capital structure

The cluster analysis of types and levels of capital yielded four types of capital structures (Table 1) described by the relative, that is, low (below‐average) and high (above‐average), volumes of capitals: (i) individuals with low economic and high cultural and social capitals (n* = *8309), (ii) those with high on both economic and non‐economic capitals (n = 8293), (iii) those with high economic capital but low combined cultural and social capitals (n = 17,556), (iv) those scoring low on both types of capitals (n = 17,185).

**Table 1:** Means and standard deviations of z‐standardised capitals for the four capital structures

	Cluster							
	High economic, low cultural and social capital		Low economic, high cultural and social capital		High economic, cultural and social capital		Low economic, cultural and social capital	
	M	SD	M	SD	M	SD	M	SD
Economic capital	0.78	0.4	−0.60	0.41	1.06	0.33	−0.97	0.37
Soc‐Cul capital	−0.30	0.39	0.60	0.42	0.96	0.42	−0.43	0.35

### Descriptive statistics

The average age of respondents at the baseline was 66 (M* = *65.68, SD* = *8.853), of whom 45.3% percent were male (n* = *25,585) and 54.7% were female (n* *=* *33,255). More detailed descriptive statistics of the sample size are presented in the table below (Table 2).

**Table 2:** Description of the sample (n, %)

	%	N
Self‐perceived health status		
Less than very good	26.3	15,975
Excellent/Very good	73.7	44,735
Used healthcare services in the last 12 months		
No	10.8	6453
Yes	89.2	53,514
Dentist visits in the last 12 months		
Yes	56.2	26,567
No	43.8	34,033
Hospital stays in the last 12 months		
Yes	15.1	51,460
No	84.6	9179
Total nights in a hospital		
Median, SD	11	16.5
Owns supplementary health insurance		
Yes	39.8	36,325
No	60.2	24,020
Child books when 10		
less than enough to fill one book shelf (<25 books)	58.7	34,902
more than enough to fill one or more bookcases (>25)	41.3	24,559
Respondents’ education		
No education	22.1	13,354
Primary education	56.1	33,867
Secondary education	17.2	10,406
University education	4.5	2713
Number of social activities in a week		
No activities	45.8	27,410
One activity	42.1	25,212
Two activities	10	6009
Three or more activities	2.1	1244
Migration status		
Not born in the country of interview	12.0	7228
Born in the country of interview	88.0	52,880

### Single and combined contribution of capitals and the likelihood of having seen/talked to a doctor

Findings from the model that adjusted for demographics, country of residence, self‐perceived health and insurance status shows evidence that all three capitals have a significant association with the probability of having seen or talked to a health professional. Those with higher economic capital had 26% higher odds of having seen a health professional (OR = *1.26,* 95% CI* = *1.12–1.42), followed by those with higher social and higher cultural capital. As expected, self‐perceived health status had the largest significant association with the use of healthcare services *(*OR = 2.97*,* 95% CI = 2.77–3.18*),* followed by gender, age and health insurance. Being female, older, in poor health and having a supplementary healthcare insurance were all associated with having higher odds of having been seen by or having talked to a health professional. Older adults living in Germany, Belgium and France had significantly higher scores in the use of healthcare services compared to the reference country (Estonia).

As for capital structure, we found that compared to those with a low volume of all capitals, those with low economic but high cultural and social capital had 29% higher odds of having seen a health professional *(*OR = 1.29*,* 95% CI = 1.17–1.43*)*. Similarly, a high volume of all capitals increased odds of having seen a health professional by 41%, (OR = 1.41, 95% CI = 1.28–1.56). Those with a high volume of economic capital, but low a volume sociocultural capital still had 16% higher odds of having seen a health professional, compared to those with low volumes of all capitals. More detailed findings are presented in Table 3.

**Table 3:** Associations of economic, cultural and social capital and of capital structure on the use of different healthcare services

Use of healthcare services	Types of capitals					
	Economic capital		Cultural capital		Social capital	
	OR	95% CI	OR	95% CI	OR	95% CI
Seen/talked to a doctor	1.26	(1.12, 1.42)	1.06	(1.06, 1.14)	1.10	(1.06, 1.14)
Dentist visits	2.95	(2.71, 3.19)	1.28	(1.24, 1.31)	1.23	(1.20, 1.27)
Overnight hospital admissions	0.79	(0.71, 0.87)	0.99	(0.96, 1.03)	0.96	(0.93, 0.99)
Length of hospital stay	0.74	(0.67, 0.81)	0.99	(0.97, 1.04)	0.91	(0.88, 0.94)

Use of healthcare services	Capital structure (ref.cat. = low volume of all capitals)					
	High volume of all capitals		Low economic, high combined cultural and social capital		High economic, low combined cultural and social capital	
	OR	95% CI	OR	95% CI	OR	95% CI
Seen/talked to a doctor	1.41	(1.28, 1.56)	1.29	(1.17, 1.43)	1.16	(1.07, 1.25)
Dentist visits	3.26	(3.03, 3.51)	1.91	(1.78, 2.04)	1.76	(1.67, 1.86)
Overnight hospital admissions	0.81	(0.74, 0.90)	0.88	(0.81, 0.96)	0.91	(0.85, 0.97)
Length of hospital stay	0.76	(0.69, 0.83)	0.86	(0.79, 0.93)	0.88	(0.83, 0.94)

Adjusted odds ratios and 95% confidence intervals from multilevel logistic regression.

Adjusted for: age, gender, marital status, country of birth, country of residence, self‐perceived health, basic insurance and supplementary insurance.

### Single and combined contribution of capitals and the likelihood of dentist visits

After controlling for demographics, perceived health and insurance, we observed that economic capital had the largest contribution to explain dentist visits (OR = 2.95, 95% CI = 2.716–3.19). Cultural capital increased the odds of having visited the dentist by 28% (OR = 1.28, 95%, CI = 1.24–1.31). For social capital, this value is 23% (OR = 1.23, 95% CI = 1.20–1.27). Those who were older, not married and rated their health as poor were significantly less likely to use dentist service in the past year. Those with supplementary health insurance and those living in Germany, Sweden and Denmark had significantly higher odds of having used dentist services.

Investigating the combined contribution of capitals, we found that compared to those with a low volume of all capitals, those with a high volume of all capitals were three times more likely to have had a dentist visit (OR = 3.26, 95% CI = 3.03–3.51), having low economic and high sociocultural capital still almost doubled the odds of accessing a dentist (OR = 1.91, 95% CI = 1.78–2.04), and having a high volume of economic capital, but low sociocultural capital, increased the odds of having visited a dentist by 76% (OR = 1.76, 95% CI = 1.67–1.1.86).

### Single and combined contribution of capital and hospital admissions

Self‐perceived health, age, gender and insurance status were significantly associated with the outcome variables. Men, those at higher ages and in poor health had higher probabilities of both hospital admission and staying more days in the hospital. Looking at the single contributions of capitals on overnight hospital stays showed a significant effect of economic capital. Here a higher volume of economic capital was associated with a decrease in overnight hospital admissions, and in the number of days spent in hospital by 22% (OR = 0.79, 95% CI = 0.71–0.87). On the other hand, social capital had a small but significant effect on having been admitted to a hospital (OR = 0.96 95% CI = 0.92–0.99), and 9% lower odds of staying more days in the hospital (OR = 0.91, 95% CI = 0.88–0.94). Cultural capital on the other hand, was not associated with overnight hospital stays, nor with the number of days spent in hospital.

Considering capital structure, we found that compared to those with low volumes of economic and non‐economic capitals, those with higher volumes of both types of capitals had 12% lower odds of being hospitalised overnight (OR = 0.88 95% CI = 0.81–0.96), and 24% lower odds of staying more days in the hospital. Those within the category of low economic, but high sociocultural capital had 19% lower odds of being hospitalised overnight (OR = 0.81 95% CI = 0.74–0.90), and 14% lower odds of staying more days in the hospital. Compared to the reference group, higher volume of economic, but lower volume of sociocultural capital was associated with 9% lower odds of overnight hospital stays (OR = 0.91, 95% CI = 0.850–0.971)*,* as well as 12% lower odds of staying more days in the hospital.

## Discussion

The analysis was conducted in two steps, starting with an investigation of the link between a particular type of capital with health care and hospital utilisation in relation to the other two capitals. In a second step, the focus shifted to an investigation of the importance of the structure of capital ownership. Through Bourdieu's notion of “habitus,” these two analytical moves clearly show that inequalities in healthcare access are rooted in the different volume and structure of capitals that in turn relate to different levels of healthcare utilisation.

After controlling for demographics (age, gender, marital status, country of birth), country of residence, perceived health and insurance, findings from the first analysis show the association of individual capitals with the use of health and hospital services. Economic capital was found to be the largest contributor of having a contact with a health professional or visiting a dentist. On the other hand, economic and social capital had a protective effect in terms of hospital admissions and the number of days stayed in the hospital. Cultural capital was strongly associated with visiting a dentist and had a small but significant contribution in seeing or talking to a health professional. However, the broader finding is that all three forms of capital play a role in the use of health, dentist and hospital services. It is possible to explain this by linking each type of capital with a particular set of practices that increase the likelihood of using health services. This article's results thus lend credence to the link between the capitals and particular health promoting practices. As noted above, those with high levels of economic capital are “free from necessity” and may develop more strategic and long‐term practices in managing their health, while those with low economic capital can afford only to satisfy their immediate needs and use healthcare services only when necessary.

It is expected then that those who do not use primary healthcare services regularly will be at an increased risk of hospital admissions, and stay longer in the hospital due to poorer health. Cultural capital is linked to particular behaviours and health habits developed since early childhood. This is in line with the Missinne *et al*. (2014) study that found that wealth and cultural capital measured through education and presence of books in childhood had a significant effect on screening practices in older age women. Those with high social capital can regularly draw on health‐related information and are embedded in support networks that can encourage healthy practices. For example, our findings revealed that in addition to facilitating access to healthcare services, social capital protects against hospital admissions and longer hospital stays. According to Longman *et al*. (2013), older populations who are socially isolated have reduced capacities to manage chronic illnesses, stress, reduced energy and motivation which can lead to more frequent hospital admissions and lengthy hospital stays.

The second analytical move at the heart of this article is the focus on capital structure in addition to the volume of each capital possessed. The cluster analysis performed on the simple binary “economic vs sociocultural capital” yielded four types of capital structures that were associated with clear differences in patterns of healthcare and hospital utilisation. This points to the empirical existence of different capital structures within the population that are associated with distinctive practices linked to the use of health and hospital services. What is particularly striking is the extent to which the hierarchy between groups is consistently the same for the different variables tested. In every case, outcomes are best for those with both high economic and high sociocultural capital, followed by those with low economic but high sociocultural capital, then those with high economic capital but low socio‐cultural capital and finally those with low volumes of all capitals. This shows that inequalities in the use of healthcare services are sensitive to immaterial, sociocultural factors and reinforces the need to go beyond economic factors when examining class inequalities in health.

Bourdieu also argues that the family plays a decisive role in the maintenance of the social order, through social as well as biological reproduction (Bourdieu 1998). For him, the family is a key site of capital accumulation and the vector through which the capitals are transmitted across generations. This is reflected in our analysis that shows that among the three capitals, economic capital measured through individual wealth was the best individual predictor of the use of healthcare services (health professional and dentist). Intergenerational transmission seems to not only play a role in the case of economic capital, but also for cultural capital: the cultural environment in which an individual was raised (Bourdieu's idea of embodied cultural capital) affects the likelihood of engaging with healthcare services. Each of these capitals, and their composition, can then be used as a resource to achieve a more optimal use of general health services and in turn lessen the likelihood of hospital admission and time spent in hospital. Thus, our findings show that it is crucial to consider capital in all its forms and structures to understand some of the class‐related practices of health and hospital services utilisation originated by the embodiment of individual's socioeconomic conditions.

Although our research showed the importance of the Bourdieusian approach to healthcare utilisation it is important to acknowledge that there were several limitations to this study. Firstly, we are aware of the limited range of variables standing for Bourdieu's concept of social capital. In this respect, it would be useful to have measures of the context of the social relationship, that is, whether the individual's social network can be drawn on to speed up health procedures or to choose the right healthcare provider and the most respected doctors. Furthermore, limited by the variables in SHARE wave five, we could not fully measure embodied cultural capital as there was no fine‐grained variable giving insight into the different cultural activities that one attends. In addition to cultural activities, variables on health behaviour have been used to measure the cultural capital reflected in group distinctive tastes such as smoking and alcohol consumption in the UK. Despite the applicability of this measure in a country‐specific context, these variables are quite problematic in cross‐country research as cultural tastes vary across countries (Bourdieu 1986). For example, a cross‐country analysis on the relation between education and health behaviour found that in Southern European countries higher educational levels are associated with a higher smoking prevalence (Avendano *et al*. 2009). Another limitation of the study arises from the rather high non‐response rate concerning information about household wealth. The currently most accepted analytic strategy is to use the averaged multiply imputed values provided by the SHARE team. Since we do not have sufficient knowledge about the non‐response mechanism that generated the missing values for wealth (Rubin 1996), and it can be suspected that missing values are not missing at random (NMAR), the analyses may still suffer from some bias. Finally, our results might hold only across a range of European countries where the vast majority of respondents enjoys universal health coverage. Caution needs to be taken with the generalisability of our results beyond the European context, especially in countries that lack universal healthcare coverage – such as in the United States for example.

In conclusion, this article highlights the importance of applying Bourdieu's theory of capitals to uncover some of the possible class‐related mechanisms that contribute to the production of health practices related to the use of health and hospital services in later life. Further longitudinal research is needed to untangle the relative importance of socio‐cultural assets which the individual receives from its broader family and social environment. It would also be important for qualitative studies to explore in more depth the links between the capitals as well as the mechanisms through which they influence the use of healthcare services. Given the different healthcare systems and welfare regimes, it is also important to explore the importance of each of the capitals and of capital structure across different health systems and welfare states.

The article has shown some of the routes through which the ownership of economic, social and cultural capital influences the use of healthcare services. Two possible conclusions for policy can be drawn from this. The first is that public authorities should encourage individuals to develop their ownership of the individual capitals. While it may be more difficult to build economic capital, developing social networks and gaining knowledge and educational qualifications could be encouraged. However, it is likely that this will be insufficient to equip individuals with the necessary resources to navigate complex health systems. This is because the social field in which the capitals grant particular advantages when it comes to health access is rigidly structured, and that what matters are relative differences in the amounts of capitals owned by individuals. In this interpretation, even if those who are “close to necessity” manage to acquire social and cultural capital, these levels of capital will not grant them the advantages enjoyed by those who are already possess high levels of these capitals. It seems to us that this interpretation is in line with Bourdieu's concern with the structuration of the social field and fits with our findings on the importance, not only of the volume of capitals, but also of their composition at the individual level. In policy terms, the implication is that interventions would have to target the conditions through which the possession of the right types of capitals facilitates the use of health services.
